# Factors contributing to under-reporting of patient safety incidents in Indonesia: leaders’ perspectives

**DOI:** 10.12688/f1000research.51912.1

**Published:** 2021-05-10

**Authors:** Inge Dhamanti, Sandra Leggat, Simon Barraclough, Taufik Rachman

**Affiliations:** 1 Department of Health Policy and Admnistration, Faculty of Public Health Universitas Airlangga, Surabaya, East Java, 60111, Indonesia; 2 Center for Patient Safety Research, Universitas Airlangga, Surabaya, East Java, 60111, Indonesia; 3 School of Psychology and Public Health, La Trobe University, Bundoora, VIC, 3083, Australia; 4 Department of Criminal Law, Faculty of Law, Universitas Airlangga, Surabaya, East Java, 60286, Indonesia

**Keywords:** under-reporting, patient safety, government organization, independent agencies, hospital, leader

## Abstract

**Background: **Understanding the causes of patient safety incidents is essential for improving patient safety; therefore, reporting and analysis of these incidents is a key imperative. Despite its implemention more than 15 years ago, the institutionalization of incident reporting in Indonesian hospitals is far from satisfactory. The aim of this study was to analyze the factors responsible for under-reporting of patient safety incidents in Indonesian public hospitals from the perspectives of leaders of hospitals, government departments, and independent institutions.

**Methods: **A qualitative research methodology was adopted for this study using semi-structured interviews of key informants. 25 participants working at nine organizations (government departments, independent institutions, and public hospitals) were interviewed. The interview transcripts were analyzed using a deductive analytic approach. Nvivo 10 was used to for data processing prior to thematic analysis.

**Results: **The key factors contributing to the under-reporting of patient safety incidents were categorized as hospital related and nonhospital related (government or independent agency). The hospital-related factors were: lack of understanding, knowledge, and responsibility for reporting; lack of leadership and institutional culture of reporting incidents; perception of reporting as an additional burden. The nonhospital-related factors were: lack of feedback and training; lack of confidentiality mechanisms in the system; absence of policy safeguards to prevent any punitive measures against the reporting hospital; lack of leadership.

**Conclusion: **Our study identified factors contributing to the under-reporting of patient safety incidents in Indonesia. The lack of government support and absence of political will to improve patient safety incident reporting appear to be the root causes of under-reporting. Our findings call for concerted efforts involving government, independent agencies, hospitals, and other stakeholders for instituting reforms in the patient safety incident reporting system.

## Introduction

Patient safety is a top priority in healthcare services. Moreover, it is also a critical policy issue as about 10% of hospitalized patients experience adverse events. ^1^ In low- and middle-income countries, an estimated 134 million adverse incidents occur among hospitalized patients every year; these incidents account for an estimated 2.6 million deaths each year. Understanding the causes of incidents provides a foundation for patient safety improvement; therefore, reporting and analysis of patient safety incidents is a key imperative. Lessons learned from the reported safety incidents can help inform interventions to prevent recurrence of similar incidents. However, this can happen only if the hospitals take responsibility for instituting safety measures and share their data at the national level. ^2^

Patient safety incident reporting systems have adopted various formats; a majority of these systems require reporting of incidents by health workers. The types of incidents that need to be reported vary in each country; these range from potential events to sentinel events. The World Health Organization (WHO) has developed a framework for reporting adverse events. ^3^ Subsequently, WHO developed a minimal information model for incident reporting systems ^4^ suitable for adoption by low-income, middle-income, and developed countries. However, the reporting rates show wide variability among countries, with some countries still struggling to implement the system.

The United Kingdom is one of the countries that have successfully implemented incident reporting. English NHS organisations reported 2,246,622 incidents or 10.3% increase on the incidents reported from April 2019 to March 2020 compared to from April 2018 to March 2019.

Another example is the Taiwan Patient Safety Reporting System which by 2019, the number of participating institutions has reached 12,491, and the cumulative number of notified cases reported from 2005 has reached 714,896.

In contrast, the number of incidents reported to the Malaysian Incident Reporting and Learning system over the past 18 years of its operation has been quite low; the number of incidents reported in the year 2016 was 2,769. However, after the implementation of national online reporting system in 2017, the number of reports showed a 105.5% increase from the preceding year.

Indonesia is the fourth most populous country in the world with an estimated population of 270 million. Only half of the country’s 2,925 hospitals are accredited by the Commission for Hospital Accreditation (CHA). The national patient safety incident reporting system was launched in 2005. There are two levels of reporting: hospital level (internal reporting) and national level (external reporting). Internal reporting comprises of written reports pertaining to any incidents occurring in the hospital, ranging from near misses to sentinel events; these incidents are required to be reported within 48 hours. External reporting refers to incident reports that have been analyzed, investigated, and reported eletronically to the National Committee.

Incident reporting is a mandatory requirement for hospital accreditation; however, the performance of the reporting system is far from satisfactory. The national level data is not publically accessible. Moreover, our previous study revealed very low rates of reporting. The total number of incidents reported in 2019 was 7,465; these incidents were reported from 334 out of the 2,877 hospitals (12%) in Indonesia. ^5^

Evaluation of the sytem also revealed some weaknesses such as the existence of punitive system, lack of confidentiality, poor timeliness of reporting, and lack of responsiveness. ^6^ The existing policies, guidelines, and regulations in Indonesia, to a large extent, do not satisfy the WHO-recommended requirements for incident reporting systems. Furthermore, there is a lack of awareness and understanding of the reporting system among officials at almost all levels. Several studies have identified the barriers that contribute to low incident reporting rates in Indonesian hospitals. ^7^^–^^9^

The high prevalence of under-reporting severely undermines the capacity of incident reporting systems to promote learning and improve patient safety. We used previous framework in identifying the factors that lead to patient safety incident reporting that consisted of organizational factors, work environment, process and system of reporting, factors related to patient safety team at hospital level, knowledge and skills of reporting personnel, individual characteristics of health care professionals, professional ethics, fear of adverse consequences, and incident characteristics. In-depth characterization of factors that contribute to under-reporting is a key imperative to improve patient safety incident reporting systems. However, despite its importance, this form of study has never been conducted in Indonesia. Therefore, we aimed to analyze the factors that contribute to under-reporting of patient safety incidents in Indonesian public hospitals based on the perspectives of leaders of hospitals, government departments, and independent institutions.

## Methods

### Study design and sample

A qualitative research methodology was adopted for this study using semi-structured interviews of key informants. The approach to phenomenology was applied, as it was intended to thoroughly explore the point of view of the leaders. A purposive sample of organizations including government departments, independent institutions, and public hospitals in the East Java Province and the capital city of Indonesia; were selected for this study. The key informants included staff members working in leadership positions at organizations involved in patient safety implementation. A total of 26 participants were approached all but one agreed to be interviewed, with a total of 25 participants from nine organizations were enrolled. The details of the participants are presented in Table 1.

**Table 1.** Type of organizations and the number of participants.

Organizations	Level	Number of participants
***Government departments***		
Indonesian Ministry of Health (IMoH)	National	2
Provincial Health Office D (PHO)	Province	2
District Health Offices at District A (A DHO)	District	2
District Health Offices at District B (B DHO)	District	2
District Health Offices at District C (C DHO)	District	1
***Independent institutions***		
National Committee on Hospital Patient Safety (the National Committee)	National	2
Commission for Hospital Accreditation (CHA)	National	2
Indonesian Hospital Association (IHA) at the national and provincial levels	National	3
***Public hospitals***		
Public hospital at District A (A Hospital)	District	3
Public hospital at District B (B Hospital)	District	3
Public hospital at District C (C Hospital)	District	3
***Total***		25

The hospitals chosen were district referral public hospitals which are required to have a functional incident reporting system (internal and external reporting) managed by the hospital patient safety team for accreditation purposes; however, none of the sampled hospitals had ever reported any incident to the national level.

### Data collection

Letters were sent to the participating organizations to solicit the names of respective key persons. Participants were the key persons that were knowledgeable about the reporting of patient safety incidents in Indonesian hospitals. Following that, we arranged an interview with their respective offices, with no other people present. The focus of the interview was to determine the potential causes of under-reporting of patient safety incidents. The questions were sent to participants prior to the interview. The first author conducted the interviews in Indonesian. The interviews were lasted from 20 minutes to one hour. All interviews were audio-recorded, transcribed, coded and managed using Nvivo 10 (NVivo, RRID:SCR_014802). The majority of those interviewed did not know the researchers personally. To ensure confidentiality, the participant’s identity was noted using initials; however, the identity of the organization was not concealed. In regard the reflexivity, the researchers, in particular the first researcher, are interested in patient safety and have performed a number of research studies on the related topic. She has spent her time at the university in Indonesia and has the necessary expertise to examine the cultural and Indonesian context relevant to the research.

### Data analysis

The transcripts were not returned to the participants, nor was feedback provided to them. The transcripts were entered into NVivo 10 and analyzed using a deductive analytic method focused on pre-defined themes derived from the research questions. The deductive approach used an organizing framework that entails coding that was conducted by two coders. The data were coded based on themes, with the initial goal of identifying certain core aspects of the data that specifically relate to the research questions. ^10^ The first step was data reduction which entailed selection of the section or text from the transcript and their coding based on the themes. The second step entailed displaying the data in tabular format followed by drawing of conclusions. Subsequently, thematic analysis was performed for the synthesis and cross-referencing of emerging topics. ^11^ We looked at the phenomenon using the principle of triangulation by looking at the phenomenon from various viewpoints, through different lenses, with different questions.

### Ethics and consent

Ethical approval for this study was obtained from the Committee on Ethics for Human Research at the Faculty of Health Sciences, La Trobe University, Australia with the ethics application number FHEC13/197. Institutional approval was also obtained from each of the participating entities. Written informed consent was obtained from respondents prior to their enrolment.

## Results

We categorized the responses according to the emerging themes. A summary of responses is presented in Table 2.

**Table 2.** Themes identified based on participant responses.

Themes	Participant responses
Benefit of reporting	- Most hospitals do not understand the benefits and the importance of reporting - Hospitals perceive no benefit of reporting
Feedback and recommendation for further action	- No direct feedback provided to hospitals - There is no guarantee that the National Committee would take corrective action based on the report
Lack of training	- Not all hospitals have received training - Lack of concern from the government regarding conducting socialization
Knowledge about reporting	- Lack of knowledge about the reporting procedure and the content of reporting - Lack of awareness among hospital staff about the need for reporting the incident - Feeling uncomfortable about reporting an incident
Confidentiality of reporting	- Many hospitals doubt the confidentiality of reporting
Consequence of reporting	- Concerns about legal issues - There is no policy to ensure that it is safe for hospitals to report incidents
The culture	- Culture of reporting has not yet been established in hospitals - There are barriers to building a patient safety culture and reporting culture in hospitals - The blaming culture within hospitals is still dominant
Reporting as a burden	- Reporting and analysis of the incident takes time and effort, especially for doctors and nurses - The hospital patient safety team’s performance is not optimal as the responsibility for carrying out the tasks or programs is assigned to a single person
The system	- Need to change the reporting system - Lack of rewards and sanctions in the system
Leadership	- Lack of leadership at the hospital level - Weak role of the IMOH in handling the reporting of incidents

Based on the data presented in the table, we identified some potential causes of under-reporting of patient safety incidents that were confirmed by the three types of organizations.

### Benefits of reporting

Participants from government departments and independent agencies agreed that the lack of appreciation of the value and significance of reporting incidents may lead to under-reporting.

“… the view from the hospital that the benefits for hospitals that report are limited, because there is no feedback.” (National Committee, A2)

“Maybe they do not understand that the goal is learning because they always ask, what's in it for us if we report?” (Indonesian Hospital Association (IHA) provincial level, A7)

### Feedback and recommendations for further action

Participants reflected on the lack of feedback provided to the reporting hospitals. This was because of the lack of annual reporting and sharing of data at the national level. As recorded by one interviewee:

“If the hospital sees that sending reports is beneficial, maybe the number of reports could increase. So that is a factor of the hospital, so in addition to internal difficulties in the hospital, the hospital also needs to be provided some kind of feedback [after reporting]” (IHA provincial level, A7)

### Lack of training

Another reported cause of under-reporting was training, as not all hospitals received training or have been socialized by the government. Some participants reported:

“Maybe because the government, such as the Ministry of Health or the Provincial health department lacks the intensity to socialize to as much detail as possible. Maybe the other reasons are afraid of being found out that the hospital is [having] bad [reputation] if they have many [reported] cases.” (C Hospital, H7)

“The cause was solely due to the government's lack of interest in socializing incident reporting.” (A Hospital, H2)

### Knowledge about reporting

Lack of knowledge was identified as one of the reasons of low reporting rates; this included the lack of knowledge about the reporting process, lack of understanding of the requirement for reporting an incident, and lack of knowledge about the anonymity of reporting. As mentioned by some participants:

“The concern from the hospital [to report the incident] was lacking” (Hospital B, H5)

“[To] Raise awareness of all health workers in this hospital to be more aware that it [the reporting] is something that needs attention.” (Hospital B, H4)

“The first cause was that health workers do not understand the importance of the reporting system. Secondly, they do not understand which incidents should be reported. (Hospital C, H8)

“But also maybe because they feel uncomfortable [in reporting] even though the report does not mention the name of the hospital, it is anonymous, but there might be inconvenience.” (IHA national level, A5)

### Confidentiality of reporting

Many participants from independent agencies emphasized concerns pertaining to the confidentiality of reporting. As some participants have remarked:

“It is their belief, [the reporting is] not confidential and so on. Convincing them is also not easy, sometimes they have made it [the internal reporting], but it is not reported to the external agency. That [...] well, that might have caused low reporting. "(National Committee, A1)

### Consequences of reporting

Some participants reported that the fear of repercussions of incident reporting, both personal and institutional, is a common cause of low reporting. According to an ICHA participant, fear of litigation by the patient often prevents the reporting of incidents attributable to acts of omission or commission by a health worker. This is due to lack of policy safeguards for the reporting hospital. As one participant reported:

“There must be some kind of law that guarantees that this report problem is safe for the hospital.” (IHA provincial level, A7)

### The culture

Hospitals are yet to institutionalize a culture of patient safety and incident reporting owing to the prevalence of a blaming culture in hospitals. Some participants reported:

“I think there are many factors that become obstacles at the hospital level, ranging from difficulties in building a culture of safety to difficulties in building a culture of reporting. (IHA provincial level, A7)

“This hospital should also not cover up what happened […] sometimes it covers up what happens.” (Hospital B, H4)

### Reporting as a burden

The participants working at hospitals claimed that incident reporting is a cause of additional stress for health workers, especially doctors and nurses. Moreover, the workload is not fairly distributed within the hospital patient safety team as only one person is usually assigned the task of incident-monitoring, reviewing, and taking further actions.

“The patient safety team itself cannot distribute the tasks, so the task is assigned to one person.” (Hospital C, H8)

### The system

The perspectives from the independent agencies highlighted the need to change the reporting system from voluntary to mandatory. One participant reported:

“So this reporting should not only be encouraged but must be made mandatory […] if not reported there must be feedback from [...] the related agencies about the lack of reporting, that is." (IHA provincial level, H2)

The participants also emphasized the need for direct feedback from the related organization, both for reporting and non-reporting hospitals. Furthermore, there is no formal system of rewards and punishment, which could help improve the reporting.

### Leadership

The participants from the independent agencies and hospitals mentioned about the lack of leadership at the government and hospital level. Strict monitoring and oversight is required for reporting of accidents, according to hospital-based participants. Moreover, hospital leaders also fail to understand the blame-free principle of incident reporting. Lastly, lack of participation by the regional health office was also one of the triggers for under-reporting.

“So indeed there must be a strict control, so frankly from the management there must be strict control, [...] that means yes [...] including supervision attached to the reported.” (Hospital B, H4)

“One of the causes for not reporting is punishment, so people do not want to report. Actually, the leader must have understood that concept of non-punitive safeguards against incident reporting?” (IHA national level, H1)

“Although the government has included [patient safety] in the accreditation standard, it needs to emphasize the involvement of regional health offices in this patient safety incident reporting system, so that several organizations that carry out monitoring can check and re-check each other” (IHA provincial level, H2)

We then classified the factors as hospital-related and nonhospital-related (government or independent agency) factors, as seen in Table 3.

**Table 3.** Categorization of the causes of under-reporting.

Hospital-related factors	Government or independent agency-related factors
Lack of understanding of the benefits of reporting	Policy-safeguards against any punitive measures against the reporting hospitals have not yet been developed
Lack of knowledge about reporting	Lack of government leadership
The responsibility to report the incident	Lack of feedback and socialization provided by related agency to hospitals
Lack of hospital-level leadership	Concerns pertaining to system confidentiality
Non-existing reporting culture	The nature of reporting should be changed from voluntary to mandatory
Reporting as an additional burden for health workers	

## Discussion

Reporting of patient safety incidents in Indonesia continues to face many challenges. Most of the causes of under-reporting identified in this study have been reported in previous studies conducted in Indonesia ^7^^,^^12^^–^^14^ and globally, ^15^^,^^16^ either as barriers to reporting of incidents or as factors that affect patient safety incident reporting. After almost two decades, the implementation of the reporting system has not reached its potential and some classical problems have continued to persist. ^11^

This study found a divergence between government departments and independent organizations on the one hand and hospitals on the other hand about the perceived causes of under-reporting. Respondents from government departments and independent organizations reported about the lack of feedback for the hospital and lack of awareness of the benefits of reporting as the causes of under-reporting; hospitals, on other hand, did not refer to the same problem. Conversely, respondents from hospitals referred to the burden of reporting which was not reported by other organizations. This discrepancy could be attributed to the fact that the sampled hospitals had never reported the incidents to the National Committee; therefore, they were not aware about the issue of lack of feedback or did not perceive the benefits of incident reporting.

Reporting of incidents is an essential first step to learn from the experience. However, in Indonesia, very little work has been done to document the lessons learned from the national patient safety incident reporting and how it can improve the processes of care or patient outcomes. There has been a lack of institutional feedback mechanism ever since the inception of the reporting system. As of April 2021, no annual reports, comprehensive information, or sharing of lessons learned from the reported incidents have been published on the website of the National Committee. This is unfortunate because lessons learned from the incidents can help save lives. Thus, many lives may have been lost just because the national system failed to learn from the incidents.

The root causes of under-reporting, either the hospital- or government-related, may reflect the lack of government support and the political will to improve patient safety incident reporting. Political will refers to the willingness of political leaders to take action to achieve a set of goals and to sustain the costs of these actions over time with some components include public commitment and resource allocation, enforcement of credible sanctions, continuity of effort, and institutionalization of learning and adaptation. For example, lack of funding for incident reporting in Indonesia was found to constrain the usefulness of reporting. ^11^ Additionally, the role of government in upgrading knowledge and skills of health workers, either through socialization or training in incident reporting, was found to be inadequate; this contributed to the lack of knowledge about the reporting procedure among health workers, lack of understanding of the benefits of reporting, and the absence of institutional reporting culture. The clear message about the importance of reporting in the national policy has not been translated into daily practice at the hospital level. As a consequence, there is a lack of reporting culture.

Additionally, there is weak enforcement of the credible sanctions regarding the implementation of internal and external reporting system by hospitals as mentioned in Standard 9 of Patient Safety and Quality Improvement. ^17^ The consequences for hospitals that fail to report or meet the quality standards and accreditation requirements have not been clearly stated; consequently, only 12% of Indonesian hospitals reported incidents in 2019. ^5^ To improve reporting, policymakers must set specific and achievable goals for the incident reporting system; for example, application of credible sanctions for hospitals that do not report their incidents, although it is one of the mandatory requirements for accreditation.

There is poor continuity of efforts for assessing, monitoring, and evaluating the incident reporting system. The hospital incident reporting systems are fragmented and isolated; in addition, establishment of best practices for implementation requires data analysis and sharing at the national level. This also reflects the failure of government to learn and adapt to the emerging circumstances through the fifteen years of the incident reporting implementation.

Reforms in patient safety incident reporting are required to help overcome the government or independent agency-related causes of under-reporting in Indonesia. These reforms should include development of a national patient safety strategic plan, setting of priorities, creating a time line, implementing the plan, monitoring and evaluation of the implementation of the policy, and revision and updation of the policy. ^18^ A good example has been shown by the Malaysian Ministry of Health. In Malaysia, patient safety incident reporting is included as one of the patient safety goals; the incident reports are compiled regularly and analyzed every three months by the healthcare facilities and submitted to the National system by by 31st January of the subsequent year. A clear, unambiguous and firm policy is required to develop a successful system. To address the confidentiality issue, Indonesia should adopt the NHS policy where in the identity of the reporter, patient, health worker, and other individuals involved in the incident is not reported. The system is programmed to remove any personal identifiers in the report. This inculcates a sense of safety among the reporting health workers and hospitals and helps increase the number of reports. Further, the reporting also needs to be categorized into mandatory reporting for adverse events and sentinel events and voluntary reporting for any other incidents. The primary focus of reporting should be to draw lessons. Reporting needs to be made compulsory and no incident should be reported as zero incident, so that there is no excuse for not reporting the incident. Lastly, good patient safety leadership at the national, local, and hospital level is crucial to foster institutional changes and improve patient safety.

A key limitation of this study is the potential lack of representativeness of the study sample. Moreover, the opinions of individuals may not be a true reflection of the organization. Thus, due diligence should be exercised while interpreting our results. However, this study addresses several critical issues related to the reporting of patient safety incidents and identifies several areas for improvement.

## Conclusion

Our study identified several causes of under-reporting of patient safety incidents in Indonesia from the perspectives of government departments, independent agencies, and hospitals. Our findings call for concerted efforts by government agencies, independent agencies, hospitals, and other stakeholders to institute comprehensive reforms in the patient safety incident reporting.

## Data Availability

OSF: Underlying data for ‘Factors contributing to under-reporting of patient safety incidents in Indonesia: leaders’s perspectives’. https://doi.org/10.17605/OSF.IO/HM7BX. ^21^ The project contains the following underlying data:
• Interview results. OSF: COREQ checklist for ‘Factors contributing to under-reporting of patient safety incidents in Indonesia: leaders’s perspectives’. https://doi.org/10.17605/OSF.IO/HM7BX. ^21^ Data are available under the terms of the Creative Commons Zero “No rights reserved” data waiver (CC0 1.0 Public domain dedication).
