# Supplementary material for: Factors contributing to under-reporting of patient safety incidents in Indonesia: leaders’ perspectives
Source: F1000Res. 2022 May 25;10:367. Originally published 2021 May 10. [Version 2] doi: 10.12688/f1000research.51912.2 (PMC9243549; doi:10.12688/f1000research.51912.2)
Supplement: Peer review history

# Peer review: Reviewer response for version 2

**DOI:** 10.5256/f1000research.134017.r139009

**Published:** 2022-06-29

**Authors:** Katie MacLure

**Affiliations:** 1 Independent Research Consultant, Aberdeen, UK

The authors have covered all the points I raised in the initial review so I find the manuscript acceptable in its current form.

Is the work clearly and accurately presented and does it cite the current literature?

Partly

If applicable, is the statistical analysis and its interpretation appropriate?

Not applicable

Are all the source data underlying the results available to ensure full reproducibility?

Yes

Is the study design appropriate and is the work technically sound?

Partly

Are the conclusions drawn adequately supported by the results?

Partly

Are sufficient details of methods and analysis provided to allow replication by others?

Partly

Reviewer Expertise:

NA

I confirm that I have read this submission and believe that I have an appropriate level of expertise to confirm that it is of an acceptable scientific standard.

# Peer review: Reviewer response for version 2

**DOI:** 10.5256/f1000research.134017.r139010

**Published:** 2022-05-26

**Authors:** Yohanes Kambaru Windi

**Affiliations:** 1 Poltekkes Kemenkes Surabaya, Surabaya, Indonesia

The statement "However if during the interview day the participant was suddenly not available, they refer to someone who was familiar with the issue". 

 Suggestion: If all targeted key informants were available for the interview. I think that the sentence above is not necessary and perhaps deleted.

 Overall the manuscript is worth reading and ready to be indexed (approved). Just add the date of ethical clearance as stated in the author's response.

Is the work clearly and accurately presented and does it cite the current literature?

Partly

If applicable, is the statistical analysis and its interpretation appropriate?

Not applicable

Are all the source data underlying the results available to ensure full reproducibility?

Yes

Is the study design appropriate and is the work technically sound?

Partly

Are the conclusions drawn adequately supported by the results?

Partly

Are sufficient details of methods and analysis provided to allow replication by others?

Partly

Reviewer Expertise:

I have a background in public health, especially health promotion and behavior, health system, health insurance, and qualitative research

I confirm that I have read this submission and believe that I have an appropriate level of expertise to confirm that it is of an acceptable scientific standard.

# Peer review: Reviewer response for version 1

**DOI:** 10.5256/f1000research.55123.r127959

**Published:** 2022-04-04

**Authors:** Yohanes Kambaru Windi

**Affiliations:** 1 Poltekkes Kemenkes Surabaya, Surabaya, Indonesia

**Abstract:**

- Delete the sentence, *“from the perspectives of leaders of hospitals, government departments, and independent institutions.”,* as it is already stated in the Method subsection.
- A brief statement on how 25 participants were recruited is important to state in the method subsection.

**Introduction:**

- There are no supporting sources of reference in paragraphs 3 to 6.
- It is stated that *“Several studies have identified the barriers that contribute to low incident reporting rates in Indonesian hospitals.”* What are they? Give an example of the reports.

**Methods:**

- Study design and sample: Explain why the participants come from the leaders, not others. If the leader then in what level? Why aren't the staff responsible for the reporting task?
- I am afraid this sentence, “ *In regard the reflexivity, the researchers, in particular the first researcher, are interested in patient safety and have performed a number of research studies on the related topic. She has spent her time at the university in Indonesia and has the necessary expertise to examine the cultural and Indonesian context relevant to the research”* goes to the introduction section as the background of this study.
- Table 2 perhaps add one column to explain who are the participants (what level of management).
- Provide the date of Ethical clearance issued.

**Results:**

- More extended quotes will be beneficial to provide a comprehensive picture of the problem of reporting system
- Confidentiality of reporting: Provide more quotes from the participants as It is stated “some participants have remarked”
- If possible, place the statement of the three types of informants (organization) for each theme.

**Discussion:**

- Supporting references to the claim of this research should be expanded.
- Detail of discussion each theme generated from the analysis will make the manuscript completer and more interesting.

**Conclusion:**

- A little expansion of the conclusion is needed.
- Refers back to the theme drawn in your results. The conclusion needs to state these issues as the contributing factors of under-reporting of a patient safety incident.
- As a factual contribution to this study, would you provide an example of reformation on patient safety incident reporting?

Is the work clearly and accurately presented and does it cite the current literature?

Partly

If applicable, is the statistical analysis and its interpretation appropriate?

Not applicable

Are all the source data underlying the results available to ensure full reproducibility?

Yes

Is the study design appropriate and is the work technically sound?

Partly

Are the conclusions drawn adequately supported by the results?

Partly

Are sufficient details of methods and analysis provided to allow replication by others?

Partly

Reviewer Expertise:

I have a background in public health, especially health promotion and behavior, health system, health insurance, and qualitative research

I confirm that I have read this submission and believe that I have an appropriate level of expertise to confirm that it is of an acceptable scientific standard, however I have significant reservations, as outlined above.

# Response

**Published:** 2022-05-15

**Authors:** Inge Dhamanti

**Affiliations:** Universitas Airlangga, Indonesia

**Abstract:**

- Delete the sentence, *“from the perspectives of leaders of hospitals, government departments, and independent institutions.”,* as it is already stated in the Method subsection.

**Response: ** Deleted as suggested

  

- A brief statement on how 25 participants were recruited is important to state in the method subsection.

**Response:**  More information has been added to the Methods section.

**Introduction:**

- There are no supporting sources of reference in paragraphs 3 to 6.

**Response:**  There are some references in paragraphs 3 to 6 in the form of link as instructed by the Guidelines for Author below:

 Web links, URLs, and links to the authors’ own websites should be included as hyperlinks within the main body of the article, and not as references.

  

- It is stated that *“Several studies have identified the barriers that contribute to low incident reporting rates in Indonesian hospitals.”* What are they? Give an example of the reports.

**Response:**  More information has been provided.

**Methods:**

- Study design and sample: Explain why the participants come from the leaders, not others. If the leader then in what level? Why aren't the staff responsible for the reporting task?

**Response: ** Leaders play critical roles in the development of an effective and efficient reporting system. We felt it was critical to understand the leader's perspective as well. We included the health workers/staff responsible for incident reporting in a separate study.

 More information is added about the level of leadership.

 Staff members in leadership positions, such as executives from independent institutions, heads of government organizations such as DHO/PHO, and hospital directors or heads of units, were among the key informants.

  

- I am afraid this sentence, “ *In regard the reflexivity, the researchers, in particular the first researcher, are interested in patient safety and have performed a number of research studies on the related topic. She has spent her time at the university in Indonesia and has the necessary expertise to examine the cultural and Indonesian context relevant to the research”* goes to the introduction section as the background of this study.

**Response:**  Thanks for your feedback. The sentences has been removed.

  

- Table 2 perhaps add one column to explain who are the participants (what level of management).

**Response:**  As the name of the organization was revealed, the position of the participants in the table provided clues as to who the participants are. The information has instead been added to the method section.

  

- Provide the date of Ethical clearance issued.

**Response:**  As this study was part of a larger study, the ethical clearance was dated 10 October 2013, with data collection taking place from 2013 to 2015.

**Results:**

- More extended quotes will be beneficial to provide a comprehensive picture of the problem of reporting system

**Response: ** Thanks for your feedback.  We've included some examples of extended quotes in the results section. Table 2, on the other hand, only highlighted the themes and summaries of the quotes.

  

- Confidentiality of reporting: Provide more quotes from the participants as It is stated “some participants have remarked”

**Response:**  Thanks for your feedback. More quotes have been added in the Result section.

  

- If possible, place the statement of the three types of informants (organization) for each theme.

**Response: ** In the result section, we have already described each theme and provided extended quotes accompanied by the type of organization. The majority of the benefits of reporting theme, for example, came from government departments and independent agencies that stated that hospitals see no benefit from reporting incidents.

**Discussion:**

- Supporting references to the claim of this research should be expanded.

**Response: **Thanks for your feedback. Some new references have been added in the Introduction and Discussion sections.

  

- Detail of discussion each theme generated from the analysis will make the manuscript completer and more interesting.

**Response: ** Thank you for your feedback. We believe there are many other literatures that focus on each theme or the factors contributing to under-reporting; however, in this paper, we want to highlight the differences in leaders' perspectives on the factors. As a result, rather than going into detail about each theme, we classified the factors into two groups and discussed the root cause and potential recommendations.

**Conclusion:**

- A little expansion of the conclusion is needed.

**Response:**  The conclusion has been revised.

  

- Refers back to the theme drawn in your results. The conclusion needs to state these issues as the contributing factors of under-reporting of a patient safety incident.

**Response: **Thanks for your suggestion. We revised the conclusion to include more information about the contributing factors.

  

- As a factual contribution to this study, would you provide an example of reformation on patient safety incident reporting?

**Response:** In the Discussion section, we provided examples of WHO-recommended reforms that could be implemented in Indonesia. A Malaysian example and the National Health System were also included.

# Peer review: Reviewer response for version 1

**DOI:** 10.5256/f1000research.55123.r92864

**Published:** 2021-09-09

**Authors:** Katie MacLure

**Affiliations:** 1 Independent Research Consultant, Aberdeen, UK

**Factors contributing to under-reporting of patient safety incidents in Indonesia: leaders’ perspectives**

 Thank you for the opportunity to review your article. It is well-written and poses important questions for the Indonesian health service and therefore patients and their families. I believe your article can be strengthened by following my constructive comments as follows. I wish you well with your manuscript.

**Abstract**

- Check grammar in the final sentence of the Methods.
- Check consistency of hyphenating hospital related.
- The sentence starting 25 participants should be moved to Results section. There should instead be a description of the interview questions in the Methods section.
- The Methods section should contain a statement on ethical review and how participants were recruited.

**Introduction**

- Sentinel events needs further explanation.
- Last sentence in paragraph 2 needs at least one reference as does many of the following paragraphs in the Introduction.
- Paragraph 4, has should be had to maintain past tense.
- It is unusual to have single sentence paragraphs so consider merging 3 and 4.
- Typo – 'sytem' instead of 'system'.
- The final paragraph of the Introduction is entirely unreferenced. This is a serious issue impacting the quality of this article.
- There is needs to be a description of tools previously used to explore lack of reporting.

**Methods **

- Need to explain why using phenomenology and what it means in this context and provide references. Which type of phenomenology? Can you make this claim when you spoke to some for as little as 20 minutes with no follow up?
- Inconsistency in calling participants key informants / leaders.
- The numbers participating should be reported in the Results not Methods section.
- No mention of informed consent nor ethical issues (found later at end of Methods section). If you report a participant’s initials and hospital name then they are in effect identifiable – did they consent to such exposure? As they were nominated by someone more senior within the hospital then their identity is known and could have repercussions. Were they provided with an information sheet?
- Need to justify the decision to share the questions in advance. Fine to do so, but you must explain and reference.
- Grammar – ‘the interviews were lasted’.
- Delete the last sentence in the Data Collection paragraph. It is irrelevant and should have been declared in third person.
- Data analysis first sentence is repetition from Data collection section.
- Two coder but did they work independently then meet to agree or worked together from the outset?
- Table 1 should be in the Results section.
- I don’t have access to the question set so cannot relate this to the themes. What was the basis for the questions? There are many validated tools which could have been adopted to allow comparison with other studies – why did you/did you not use such a tool?
- Last sentence in Data analysis needs more explanation – which lenses? How can you apply different questions from those in the interview? You are adopting an unusual approach to triangulation so must justify and reference.

**Results**

- Are the ‘participants’ responses’ in Table 2 verbatim quotes? These are short, do they capture the context for reporting?
- Did you do a subgroup analysis of different hospital types and described earlier?
- What is the value of Table 2 being placed before the longer participant quotes? If not including verbatim quotes then it may make more sense to move to after the extended quotes.
- Tables should appear close after the statement which references the table.

**Discussion**

- ‘Updation’ is not an English word – maybe it should be!
- The Discussion section needs a lot more referencing to the related literature throughout. At points it comes across as a personal mission with relating the results to the literature.

**Conclusion**

- This is very brief. I suggest going back to your original aim and reflecting that in this section. Also indicating any further research which would support development of an improved reporting culture.

Is the work clearly and accurately presented and does it cite the current literature?

Partly

If applicable, is the statistical analysis and its interpretation appropriate?

Not applicable

Are all the source data underlying the results available to ensure full reproducibility?

Yes

Is the study design appropriate and is the work technically sound?

Partly

Are the conclusions drawn adequately supported by the results?

Partly

Are sufficient details of methods and analysis provided to allow replication by others?

Partly

Reviewer Expertise:

Digital Health; Health Inequalities; Qualitative Research

I confirm that I have read this submission and believe that I have an appropriate level of expertise to confirm that it is of an acceptable scientific standard, however I have significant reservations, as outlined above.

# Response

**Published:** 2022-05-15

**Authors:** Inge Dhamanti

**Affiliations:** Universitas Airlangga, Indonesia

**Abstract**

- Check grammar in the final sentence of the Methods.

**Response:** Thanks for your feedback.

 It has been revised into:

 The interview transcripts were analyzed using a deductive analytic approach. Nvivo 10 was used for data processing prior to thematic analysis.

  

- Check consistency of hyphenating hospital related.

**Response: **Changes have been made  

  

- The sentence starting 25 participants should be moved to Results section. There should instead be a description of the interview questions in the Methods section.

**Response: **The sentence has been relocated to the Result Section.

  

- The Methods section should contain a statement on ethical review and how participants were recruited.

**Response: **how the participants were recruited were added.

**Introduction**

- Sentinel events needs further explanation.

**Response: **Further explanation was added.

  

- Last sentence in paragraph 2 needs at least one reference as does many of the following paragraphs in the Introduction.

**Response: **Reference has been added.

  

- Paragraph 4, has should be had to maintain past tense.

**Response:** Changed into:

 Indonesia is the world's fourth most populated country, with an estimated 270 million people. The Commission for Hospital Accreditation (CHA) accredited only half of the country's 2,925 hospitals. In 2005, the national patient safety incident reporting system was established. There were two reporting levels: hospital-level (internal reporting) and national-level (external reporting). Internal reporting required written reports of all incidents that occurred within the hospital, from near misses to sentinel events; these incidences were to be reported within 48 hours. External reporting referred to incident reports that have been reviewed, investigated, and forwarded to the National Committee via electronic means.

  

- It is unusual to have single sentence paragraphs so consider merging 3 and 4.

**Response**: the paragraphs were merged

  

- Typo – 'sytem' instead of 'system'.

**Response: **revised

  

- The final paragraph of the Introduction is entirely unreferenced. This is a serious issue impacting the quality of this article.

**Response: **Reference was added to the paragraph.

  

- There is needs to be a description of tools previously used to explore lack of reporting.

**Response:** The tool is explained in the paragraph.

 We used London protocol framework in identifying the factors that lead to patient safety incident reporting that consisted of patient factors, task and technology factors, individual factor, team factor and work environment.

**Methods**

- Need to explain why using phenomenology and what it means in this context and provide references. Which type of phenomenology? Can you make this claim when you spoke to some for as little as 20 minutes with no follow up?

**Response: **Thanks for your feedback. We revised the methodology and chose qualitative descriptive analysis rather than phenomenology as the basis for our findings. Due to the fact that it is more appropriate than phenomenology.

 This was a descriptive qualitative study using semi-structured interviews of key informants that was intended to thoroughly explore the point of view of the participant.

  

- Inconsistency in calling participants key informants / leaders.

**Response: **Changed from key informants/leaders into participant.

  

- The numbers participating should be reported in the Results not Methods section.

**Response:** Table 1 has been moved into Result section

  

- No mention of informed consent nor ethical issues (found later at end of Methods section). If you report a participant’s initials and hospital name then they are in effect identifiable – did they consent to such exposure? As they were nominated by someone more senior within the hospital then their identity is known and could have repercussions. Were they provided with an information sheet?

**Response: **We sent the information sheet, informed consent form, and question list a few days before the interview. We discussed informed consent prior to beginning the interview, and once everything was clear, we began the interview. 

  

- Need to justify the decision to share the questions in advance. Fine to do so, but you must explain and reference.

**Response: **We decided to share the question ahead of time so that the interviewee would feel at ease and familiar with the subject of the interview. And I've inserted the reference.

  

- Grammar – ‘the interviews were lasted’.

**Response: **revised into “the interviews lasted …..”

  

- Delete the last sentence in the Data Collection paragraph. It is irrelevant and should have been declared in third person.

**Response:** Thanks. The sentence is removed.

  

- Data analysis first sentence is repetition from Data collection section.

**Response: **The sentence is removed.

  

- Two coder but did they work independently then meet to agree or worked together from the outset?

**Response:** Yes. The sentence has been revised.

  

- Table 1 should be in the Results section.

**Response:** Table 1 has been moved to Results section

  

- I don’t have access to the question set so cannot relate this to the themes. What was the basis for the questions? There are many validated tools which could have been adopted to allow comparison with other studies – why did you/did you not use such a tool?

**Response: **Thanks for the question. I agree that there has been a lot of research done on similar topics. However, in order to make the interview questions more relevant to the Indonesian context, we decided to develop them in accordance with the National Guidelines for Patient Safety Incident Reporting. The introduction now includes a reference to the guidelines.

  

- Last sentence in Data analysis needs more explanation – which lenses? How can you apply different questions from those in the interview? You are adopting an unusual approach to triangulation so must justify and reference.

**Response: **Thanks for the comment. What I mean by different questions is that the interview was semi-structured, and the probe to the next question could be different because hospitals, government departments, and independent institutions all have different roles when it comes to incident reporting. However, I revised the sentence to make it clearer.

**Results**

- Are the ‘participants’ responses’ in Table 2 verbatim quotes? These are short, do they capture the context for reporting?

**Response: **Table 2 did not contain the quotes but rather summary of the quotes instead. Some quotes are presented in the next paragraphs.

  

- Did you do a subgroup analysis of different hospital types and described earlier?

**Response: **As shown in table 2, all hospitals are of the same type: public hospitals. As a result, we did not conduct a subgroup analysis of different hospital types.

  

- What is the value of Table 2 being placed before the longer participant quotes? If not including verbatim quotes then it may make more sense to move to after the extended quotes.

**Response:** Thanks for your suggestion. Table 2 is removed after the extended quotes.

  

- Tables should appear close after the statement which references the table.

**Response:** Revised.

**Discussion**

- ‘Updation’ is not an English word – maybe it should be!

**Response:** Sorry for the typo. It should be updating. Revised into: These reforms should include developing a national patient safety strategic plan, establishing priorities, developing a timetable, implementing the plan, monitoring and evaluating policy implementation, and revising and updating the policy.

  

- The Discussion section needs a lot more referencing to the related literature throughout. At points it comes across as a personal mission with relating the results to the literature.

**Response:** Some references have been added

**Conclusion**

- This is very brief. I suggest going back to your original aim and reflecting that in this section. Also indicating any further research which would support development of an improved reporting culture.

**Response:** Thank you for your suggestion. We have revised the conclusion.
